# Institutional Needs

**Published:** 1918-04-13

**Authors:** 


### Institutional Needs.

### THE ECLIPSE HOT-WATER BOTTLE.

This bottle, of which we have a specimen before us, shows some good features which should commend it for institutional use. Its rubber-covered stopper overcomes all risk of losing the washer or getting it out of order, and renders leakage from this source impossible, while the broad solid seating when the stopper is screwed down renders it absolutely water-tight. The price (from 5s. 6d. upwards) is eminently reasonable, and the rubber is of good quality. The makers are J. G. Ingram and Son, London India-rubber Works, Hackney Wick, E. 9.

☛ Previous articles appeared Feb. 24, March 10, 24, 31, April 14 and 21, May 5, 12, and 19, June 9 and 16, Sept. 1 and 29, and Feb. 23, p. 445.

